# Enhancing the Thermal Stability of Ionogels: Synthesis and Properties of Triple Ionic Liquid/Halloysite/MCC Ionogels

**DOI:** 10.3390/molecules26206198

**Published:** 2021-10-14

**Authors:** Olga V. Alekseeva, Valeriya D. Shibaeva, Andrew V. Noskov, Vladimir K. Ivanov, Alexander V. Agafonov

**Affiliations:** 1 G.A. Krestov Institute of Solution Chemistry, Russian Academy of Sciences, 153045 Ivanovo, Russia; ova@isc-ras.ru (O.V.A.); leric2009@yandex.ru (V.D.S.); ava@isc-ras.ru (A.V.A.); 2 Kurnakov Institute of General and Inorganic Chemistry, Russian Academy of Sciences, 117901 Moscow, Russia; van@igic.ras.ru

**Keywords:** ionic liquid, halloysite, microcrystalline cellulose, glass-transition temperature, thermal stability, conductivity

## Abstract

In this study, an ionic liquid (IL), 1-butyl-3-methylimidazolium acetate, was used to prepare ionogels with microcrystalline cellulose (MCC) and halloysite (Hal). SEM, XRD, TG, DSC, FTIR spectroscopy, conductometry and mechanical tests were used to study the morphology, structure, thermal behaviour and electrophysical and mechanical characteristics of synthesised ionogels. XRD analysis showed a slight decrease in the interlayer space of halloysite in ionogels containing MCC, which may have been associated with the removal of residual water molecules resulting from hydrophilic IL anions and polymer macromolecules. A change in conductivity and glass-transition temperature of the ionic liquid was revealed due to intercalation into halloysite (a confinement effect) and modification with cellulose. For triple IL/Hal/MCC ionogels, the characteristic thermal degradation temperatures were higher than the corresponding values for IL/Hal composites. This indicates that the synthesised IL/Hal/MCC ionogels are characterised by a greater thermal stability than those of IL/Hal systems.

## 1. Introduction

In recent years, in light of the development of environmentally friendly processes and technologies, there has been an increasing interest in new classes of compounds, such as ionic liquids (ILs), which provide an alternative to traditional solvents [1,2,3,4,5]. Ionic liquids are molten organic salts that remain in a liquid state over a wide temperature range and have a molecular-ionic nature in a relatively large fraction of the ionic component. Due to their specific properties (low melting point, ability to dissolve organic compounds, high polarity, thermal and chemical stability, ionic conductivity, incombustibility and nontoxicity), ILs are used in various industries, as well as in biological and biochemical processes [6]. Of particular interest is the use of ILs in catalysis, electrochemical analysis and as electrolytes for fuel cells [7]. Ionic liquids are capable of absorbing and dissolving gases such as CO_2_ and O_2_. This allows ionic liquids to be used in electrochemical sensors as composite materials (conductive gels and semipermeable membranes) [5,6,7].

Le Bideau et al. [8] noted that for practical material applications, there is a challenging need for immobilizing ILs in solid matrices, while keeping their specific properties. In this regard, ionogels can be presented as a new class of hybrid materials, in which the properties of the IL are hybridised with those of another component, which may be organic (low-molecular-weight gelator, biopolymer), inorganic (e.g., carbon nanotubes, silica, etc.) or hybrid organic–inorganic (e.g., polymer and inorganic fillers).

Importantly, ionogels can easily be modified with biopolymers (such as cellulose, chitin, chitosan, lignin, etc.), as ILs are known to dissolve them [9,10,11,12,13]. Cellulose is the most abundant, easily renewable, biodegradable, nontoxic and biocompatible linear polysaccharide in nature, consisting of glucose units linked through β-(1–4) glycosidic linkages. Ionogels made from ILs and cellulose have attracted much attention, due to the possibility of creating electrically conductive materials with improved mechanical strength, elasticity and electrical conductivity comparable to that of pure ionic liquids, which make them promising matrices for collecting and storing energy and making soft artificial muscles. Lee et al. [14] developed multiphase materials consisting of cellulose nanocrystals and superbranched polymer ionic liquids. The resulting ionogels had excellent ionic conductivity (≈7.8 mS cm^−1^), close to that of traditional solid electrolytes, and showed high compression resistance (≈5.6 MPa).

Nevstrueva et al. [12] dissolved cellulose in 1-ethyl-3-methylimidazolium acetate and synthesised ionogels (CEL-iGEL) that have great potential for use in soft artificial muscles due to their flexibility, low driving voltage and biocompatibility. In an electromechanical evaluation, the CEL-iGEL actuators showed an exponential dependence of the strain difference on the voltage applied, which reached 0.6% at 2 V. Electrochemical analysis confirmed the good stability of the CEL-iGEL actuators and determined the safe working voltage value to be below 2.5 V.

One of the most widely used cellulose derivatives is microcrystalline cellulose (MCC). It is a white, fine, odourless crystalline powder possessing biocompatibility and biodegradability and having great mechanical strength, a large specific surface area and a low density. Due to its properties, MCC is widely used in the food, cosmetic, medical and pharmaceutical industries as a moisture stabiliser, emulsifier and filler for medical tablets and in suspensions, ointments and creams [15]. Recently, the use of MCC in solid polymer electrolytes has also been reported [16].

For use in ionogels, ionic liquids, such as tertiary amine N-oxides, are good cellulose solvents, the most promising ones being imidazolium and pyridinium salts. According to recent reports [12,17], the nature, size and polarisability of cations and anions are of great importance to the ability of IL to dissolve cellulose.

It should be noted that IL/biopolymer ionogels are characterised by low values of the modulus of elasticity and strength. Guo et al. [18] reported that, in order to improve the physicomechanical properties and thermal stability of ionogels, various inorganic nanofillers can be added into the polymer matrix.

Among the inorganic fillers commonly used to fill polymers, halloysite (Hal) has received much attention, since it is a natural, low-cost clay mineral consisting of multilayer aluminosilicate nanotubes with a cavity inside. Halloysite is an inert, biocompatible aluminosilicate and is chemically identical to kaolin. The general formula of halloysite is Al_2_Si_2_O_5_(OH)_4_×nH_2_O, where n is equal to 0 or 2 for dehydrated and hydrated Hal, respectively. Its external surface is composed of siloxane groups (Si–O–Si). Hydroxyl groups are located between the layers and on the outer and inner surfaces of the nanotubes. The presence of hydroxyl groups allows for the formation of hydrogen bonds between the halloysite and the polymer matrix.

The purpose of the present work was to obtain ecofriendly IL/Hal/MCC ionogels using an ionic liquid as a solvent and to study the morphology, structure and physicochemical properties of the resulting materials. Specific attention was paid to establishing how the introduction of cellulose affects the thermal behaviour and electrical conductivity of the ionogels composed of ionic liquids entrapped by halloysite. Note that the aforementioned paper [18] does not provide information on the effect of cellulose concentration on the thermal stability of the ionogels under study.

## 2. Results and Discussion

### 2.1. Surface Morphologies of Halloysite Clay and Synthesised BMImAc/Hal/MCC Ionogels 

**Figure 1:**
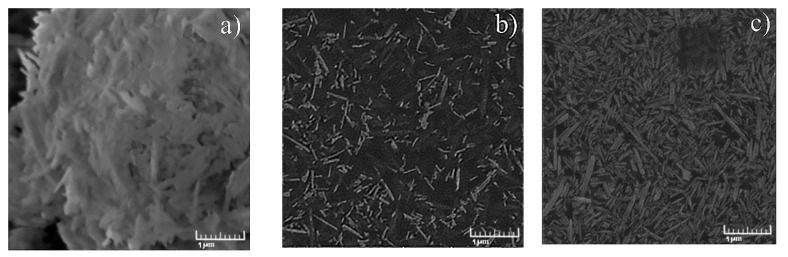
SEM image of Hal (**a**), BMImAc/Hal ionogel (**b**) and BMImAc/Hal/MCC-4 ionogel (**c**).

In this work, triple BMImAc/Hal/MCC ionogels with different concentrations of cellulose were prepared. The surface morphology of starting halloysite powder, IL/Hal and IL/Hal/MCC composites was studied using SEM, and the results are presented in Figure 1a–c. Figure 1a confirms that the halloysite nanoparticles were of cylindrical shape and were of different sizes (up to 1 μM in length). Figure 1b shows that the BMImAc/Hal composite was an ionic liquid filled with aggregated halloysite particles.

The surface morphology of the BMImAc/Hal/MCC-4 ionogel (containing 4 wt.% of MCC) is presented in Figure 1c. It is well known that microcrystalline cellulose consists of microfibrils of various lengths and a thickness of about 10–25 µm [19]. Aggregates of cellulose microfibrils and halloysite nanotubes of different sizes (0.1–1 μM) are easily discernible in the image. The introduction of MCC contributes to a more uniform distribution of the halloysite particles and, thereby, increases the viscosity of the ionogel (Supplementary Materials).

### 2.2. Crystal Structure of Halloysite Clay and BMImAc/Hal/MCC Ionogels

**Figure 2:**
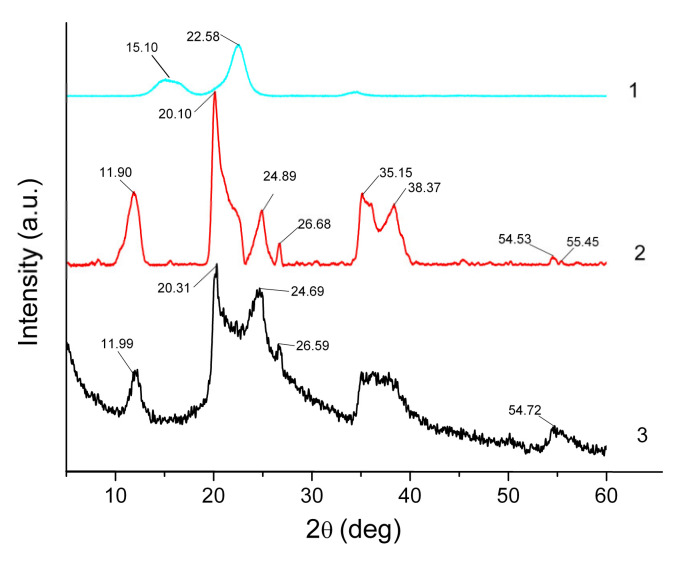
XRD patterns of MCC (*1*), Hal (*2*) and IL/Hal/MCC-1 (*3*) ionogels.

XRD analysis provides valuable information on structural changes occurring during ionogel formation. The corresponding patterns for Hal, MCC and IL/Hal/MCC ionogels are shown in Figure 2.

Figure 2 (pattern 1) shows the diffractogram of the MCC sample. The peaks at 2θ = 15.10 and 22.58° are easily discernible; the diffractogram coincides with those reported earlier for MCC samples [19,20].

The diffractogram of starting halloysite powder exhibits a reflection at 2θ=11.90°, corresponding to the basal distance (d_001_) of 0.743 nm (Figure 2, pattern *2*). This value is close to that reported by Zhang et al. [21] (0.734 nm) for halloysite in a dehydrated form, Al_2_Si_2_O_5_(OH)_4_. Other characteristic peak positions at 2θ=20.10°, 24.89°, 35.15°, 38.37° and 54.53° corresponded to crystallographic planes (020), (002), (130), (131) and (114), which also agrees well with the existing data [22,23,24,25]. The reflections at 2θ=20.10° and 24.89° indicate that the presence of quartz and two diffraction peaks at 2θ=35.15°, 38.37° may be ascribed to calcite admixture [22]. The notable broadening of the halloysite reflections can be attributed to the small size of crystallites. 

**Figure 3:**
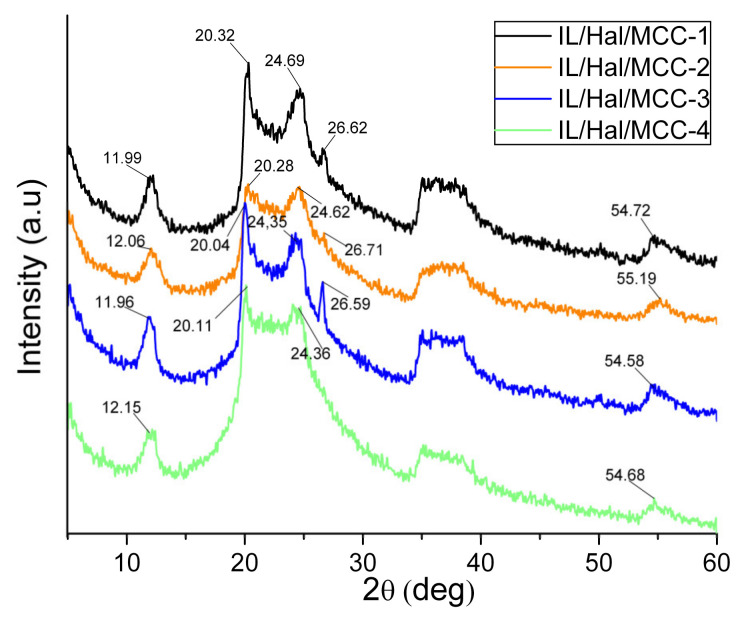
XRD patterns of IL/Hal/MCC ionogel samples.

The peaks ascribed to halloysite are also observed in the pattern of the IL/Hal/MCC ionogels (Figure 2, pattern *3*). However, in the region of 20–25° the appearance of the diffractogram is changed due to the intense peak of MCC. This change in the XRD pattern is more pronounced in ionogels with a higher MCC concentration (Figure 3).

Table 1 shows the position (2θ_0_) of the main halloysite peak (001) for ionogels with different MCC concentrations. The data presented indicate a slight decrease in the basal distance d_001_ in the clay mineral. It can be assumed that the decrease in the interlayer space is probably associated with the removal of residual water molecules due to the hydrophilic nature of IL anions and the polymer macromolecules [26].

### 2.3. Phase Transitions in IL/Hal/MCC Ionogels

**Figure 4:**
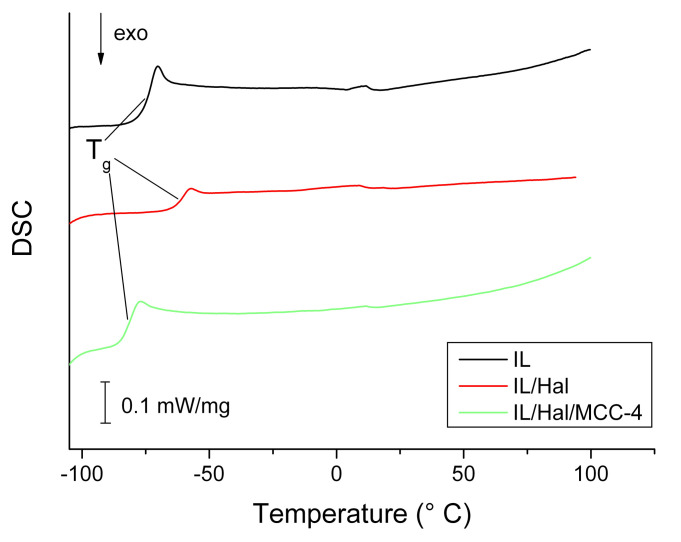
DSC data for BMImAc ionic liquid and BMImAc/Hal/MCC ionogels.

DSC curves of the second heating cycles of BMImAc and BMImAc/Hal/MCC ionogels are presented in Figure 4. 

The phase transformations caused by glass-transition processes can be observed in the low-temperature region (from −60 to −80 °C). Based on the analysis of thermograms, the following characteristic temperatures were found: the glass-transition temperatures (T_g_) corresponding to the inflection points; and the temperatures of onset and end of phase transition (*T*_onset_ and *T*_end_ values, respectively), which were determined by the tangent intersection method. 

The *T*_onset_, *T*_g_ and *T*_end_ values for the BMImAc/MCC ionogels, which were precursors for the synthesised BMImAc/Hal/MCC composites (Section 2.2), are listed in Table 2. The analysis of these data shows that for all of the characteristic temperatures there was a nonmonotonic dependence on MCC concentration, with a maximum at 2 wt. % of polymer.

Comparing the data presented in Table 2 and Table 3, one can see that, for the IL/Hal ionogel (without MCC), the glass-transition temperature was 12 °C higher than that for pure IL. A similar increase in the *T*_g_ value (by 4 °C) was observed for the IL/Hal ionogels based on 1-butyl-3-methylimidazolium dicyanamide [26]. This indicates a confinement effect, i.e., a change in the properties of ILs upon intercalation into the clay. However, when MCC was introduced into the BMImAc/Hal ionogel, the difference in glass-transition temperatures decreased. Moreover, for the IL/Hal/MCC-4 composite, lower characteristic phase-transition temperatures were observed compared to those for the composites without polymer (Table 3). It should be noted that the confinement effect is caused by the strong interaction of the ionic liquid with the inner surface of halloysite nanotubes [6,26,27,28].

### 2.4. Thermal Degradation of the IL/Hal/MCC Ionogels and Their Components

**Figure 5:**
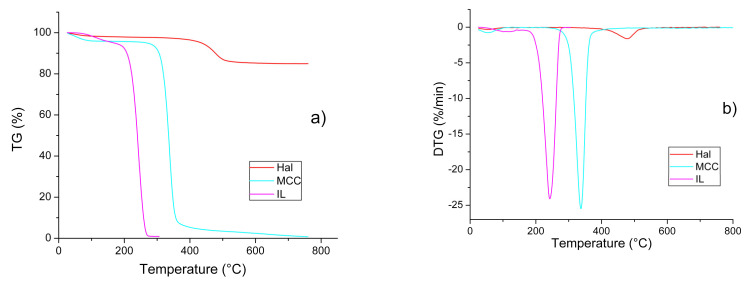
TG (**a**) and DTG (**b**) patterns for the individual components of the IL/Hal/MCC ionogels.

Figure 5a,b shows TG and DTG patterns of the individual components of the IL/Hall/MCC composites. The thermal degradation of each of the starting materials occurred in two stages. The first stage was associated with the removal of water from the samples (Δm_1_=2−6%). The onset (*T*_1_) and end (*T*_2_) temperatures of this stage were determined using the tangent intersection method. Table 4 shows that, for IL, the *T*_1_ and *T*_2_ values were 30–50° higher than those of both halloysite and polymer. This confirms the strong interaction of water and ionic liquid. 

At the second stage, a sharp decrease in the weight of the sample was observed, which was caused by thermolysis of the material. Figure 5a,b shows that, at this stage, the thermal behaviour of IL, Hal and MCC was significantly different. The ionic liquid and cellulose decomposed almost completely, while for the clay mineral the weight loss was only 13% when heated to 800 °C.

**Figure 6:**
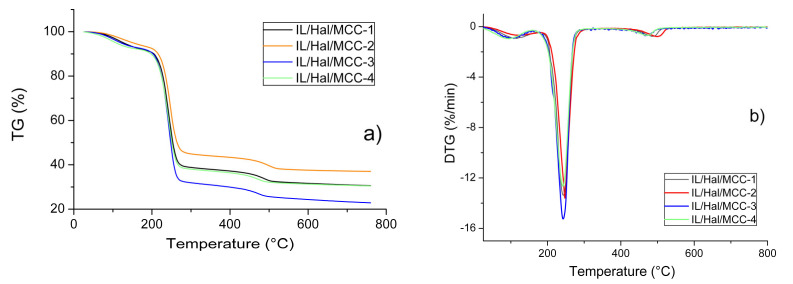
TG (**a**) and DTG (**b**) patterns for IL/Hal/MCC ionogels.

The TG and DTG patterns recorded for the BMImAc/Hal/MCC ionogels, with various concentrations of MCC, are presented in Figure 6a,b. Figure 6a demonstrates three stages of thermal decomposition of the composites. In the temperature range of 63–153 °C (first stage), water was removed from the samples. This temperature range is much wider than that of the individual components of the composites. A similar effect was reported earlier, for montmorillonite/IL systems [2].

Comparing the data presented in Table 4 and Table 5, it can be concluded that the temperature range corresponding to the second stage of thermal decomposition of the composites (217–263 °C) was close to that of IL (220–260 °C). In this temperature range, 50–60% of the composite material decomposed. During the third stage (437–524 °C), the weight of the composite decreased by 7–8%. This was connected to the thermal degradation of halloysite.

It was recently shown [26] that imidazolium ILs (such as BMImDCA, BMImOtf and BMImTFSI) confined in a halloysite matrix decomposed more readily than bulk ILs. The data presented in Table 4 and Table 5 indicate that this conclusion is also true for the BMImAc studied in the current work. Specifically, in terms of the thermal decomposition of the BMImAc/Hal composite (Table 5), the characteristic temperatures of the second stage (*T*_1_, *T*_d_ and *T*_2_) were lower than those for pure BMImAc (Table 4).

This tendency, however, does change when switching to IL/Hal/MCC composites. Table 5 shows the characteristic temperatures determined for the first, second and third stages of thermal decomposition of the IL/Hal/MCC ionogels. The presence of cellulose in the composites affected the characteristic temperatures of all of the stages of thermal destruction. For the IL/Hal/MCC ionogels, the *T*_1_, *T*_d_ and *T*_2_ values were higher than the corresponding values for the IL/Hal composites. This is of great practical importance. Replacing ionogels with similar ones with cellulose can lead to an increase in the thermal stability of the equipment in which such materials are used. 

Recently, Guo et al. [18] reported on the thermal stability of the triple ionogels similar to the object of the current study. They found that the thermal decomposition curves of the ionogels were shifted towards higher temperatures compared to those of the initial IL. However, this paper (in contrast to the current study) did not provide any data on the effect of cellulose concentration on the thermal stability of the ionogels. In addition, Guo et al. [18] ignored the possible influence of epoxy resin and cerium ammonium nitrate, which were used in their synthesis protocol.

### 2.5. FTIR Spectra of the IL/Hal/MCC Ionogels and Their Components

**Figure 7:**
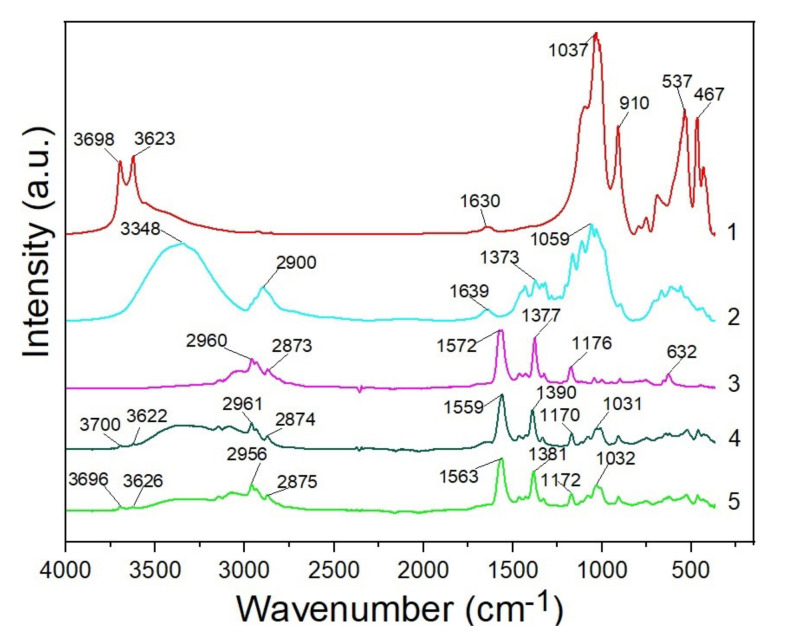
FTIR spectra of halloysite (*1*), MCC (*2*), BMImAc (*3*), IL/Hal (4) and IL/Hal/MCC-4 ionogel (5).

The FTIR reflection spectra (4000–400 cm^−1^) of the initial halloysite and MCC powders, pure BMImAc, IL/Hal and IL/Hal/MCC ionogels are shown in Figure 7. In the FTIR spectrum of the halloysite clay mineral sample (spectrum *1*), the peaks at 3698 and 3623 cm^−1^ were due to the stretching vibrations of the OH groups of the Al-OH. The band at 1630 cm^−1^ could be ascribed to the deformation vibrations of adsorbed H_2_O molecules. The band at 1037 cm^−1^ could be attributed to the stretching vibrations of Si–O groups in halloysite. The peak at 910 cm^−1^ corresponded to the bending vibrations of the Al−OH. The peaks at 537 and 467 cm^−1^ corresponded to the deformation vibrations of the Al-O-Si and Si–O–Si groups, respectively [29].

In the spectrum of microcrystalline cellulose, a wide band at 3348 cm^−1^ and a band at 1639 cm^−1^ were present, corresponding to the stretching and bending modes of the surface OH groups. The peak at 2900 cm^−1^ belonged to the asymmetrically stretching vibrations of C-H bonds in polysaccharides. The band at 1059 cm^−1^ could be attributed to C-O groups of cellulose. The bands in the range of 1638–1373 cm^−1^ could be attributed to the deformation vibrations of the CH_2_ and CH groups of the polymer. The absorption bands typical of cellulose were also observed in the range of 1639–900 cm^−1^ [30].

The IR spectrum of the BMImAc ionic liquid (Figure 7, spectrum *3*) contains all the characteristic bands specific to the BMImAc functional groups. The bands at 2960 and 2873 cm^−1^ correspond to the asymmetric and symmetric stretching vibrations (C–H), respectively [31]. Characteristic peaks (at 1572 and 1377 cm^−1^) could be attributed to asymmetric and symmetric stretching vibrations of the COO-group, respectively. The band at 1176 cm^−1^ corresponded to the stretching vibrations of the imidazolium ring. The peak at 632 cm^−1^ is associated with bending vibrations of the ring [32].

In the spectrum of the IL/Hal ionogel (Figure 7, spectrum *4*), bands could be observed that were characteristic of both the functional groups of BMImAc and clay minerals. When comparing the IR spectra of the IL/Hal/MCC composite and halloysite, one can see that, in the ionogel spectrum (spectrum *5*), the intensity of the stretching vibrations of OH groups in the range of 3700–3600 cm^−1^ was lower, indicating a decrease in the concentration of hydrogen bonds.

Analysis of the IL and IL/Hal/MCC spectra showed that, in the composite spectrum, bands at 2956 and 2875 cm^−1^ corresponded to asymmetric and symmetric stretching vibrations (C-H), as well as to stretching vibrations of the COO-group of an ionic liquid (1563 and 1381 cm^−1^), with a bathochromic shift (by 9 cm^−1^) and a hypsochromic shift (by 4 cm^−1^), respectively. A shift of the stretching vibrations band of the imidazolium ring (1176 cm^−1^) to longer wavelengths, by 4 cm^−1^, was also registered. This band and the band of the C-O group in MCC (1059 cm^−1^) were overlapped by a band corresponding to the stretching vibrations of Si-O groups in halloysite 1037 cm^−1^, which shifted to longer wavelengths, by 5 cm^−1^.

Taking into account the structure of the ionic liquid (positively charged imidazolium ring and acetate anion), it can be assumed that, during the synthesis of the composite, the donor–acceptor interaction of the imidazolium ring with the electron-donor groups of halloysite and cellulose occurred.

### 2.6. Ionic Conductivity of the Studied Materials

In recent papers [26,33], the electrical conductivity electrophysical parameters of several IL/clay ionogels have been reported, with the conclusion that the specific conductivity (*κ*) for the IL entrapped by clay differed from that of the bulk IL. Therefore, it is interesting to study the electrical conductivity of IL/clay ionogels that are further modified with a polymer. For this purpose, the conductivity of BMImAc/Hal/MCC-2 ionogel, which exhibits the greatest thermal stability, was measured.

**Figure 8:**
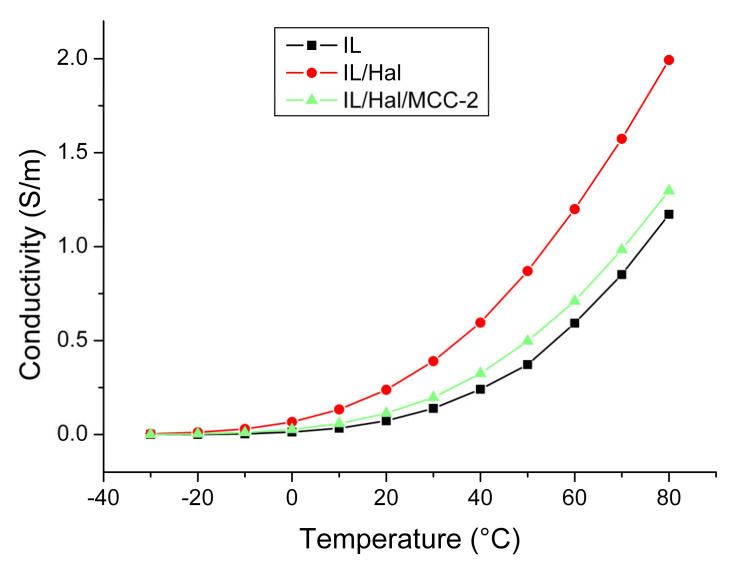
Temperature dependence of electrical conductivity for BMImAc, BMImAc/Hal and BMImAc/Hal/MCC-2.

Figure 8 shows the temperature dependences of the specific conductivity of BMImAc and ionogels based on it (BMImAc/Hal and BMImAc/Hal/MCC-2). The electrical conductivity of all investigated materials increased monotonically with temperature, which is typical of materials with ionic conductivity. The *κ* value increased by three to four orders of magnitude when the temperature increased from –30 to 80 °C (Table 6).

Figure 8 shows that the BMImAc/Hal ionogel possessed a higher electrical conductivity than pure IL. This is another consequence of the confinement effect. The higher conductivity of the BMImAc/Hal ionogel may be due to the fact that BMImAc absorbed water from halloysite, which led to an increase in the dissociation degree and mobility of ions. However, when cellulose was introduced into the BMImAc/Hal ionogel, some of the water molecules were bound to the polymer through hydration, which led to a decrease in conductivity (Figure 8). In addition, the drying of the BMImAc/Hal/MCC ionogel at 80 °C for 24 h (Section 2.2) also led to the removal of water; therefore, the confinement effect was greatly reduced.

Thus, the modification by cellulose affected the vector of changes in the electrical conductivity of ionic liquids entrapped by halloysite.

Interestingly, in contrast to our results, Guo et al. [18] reported a lower electrical conductivity of the triple ionogels compared to the initial IL (1-ethyl-3-methyl imidazole acetate (EMImAc)). The authors explained this fact by the lower concentration of ions in the ionogel, while the decrease in conductivity can also be caused by the presence of epoxy resin, which reduces the mobility of ions in the ionogel.

### 2.7. Mechanical Test Results for Synthesised Ionogels

**Figure 9:**
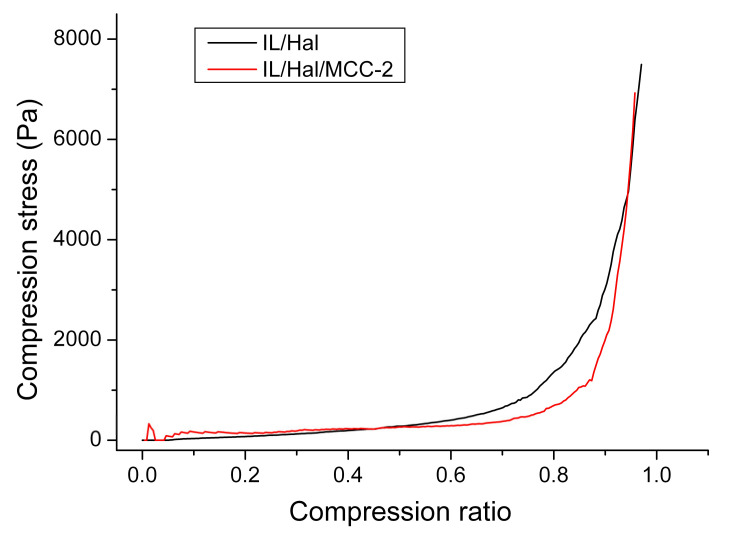
Dependence of the compression stress on the degree of deformation for the IL/Hal and IL/Hal/MCC ionogels.

The results of a study of the mechanical properties of ionogels show that, under compression, ionogels undergo a process of linear deformation under a load that is constantly increased. Figure 9 shows that the areas of linear dependence of the stress under compression versus the degree of deformation were considerably extended for both IL/Hal and IL/Hal/MCC ionogels. Young’s modulus, calculated from the slope of the straight section, was 350 Pa for the IL/Hal ionogel and 350 Pa for the IL/Hal/MCC ionogel. After reaching the compression ratio of 0.5, the linear sections of the deformation curves pass into the nonlinear growth region associated with the onset of the strain hardening process. Further compression (up to 0.85–0.95 compression ratio) led to a rapid increase in compression stress (Figure 9). 

### 2.8. The Advantages of BMImAc/Hal/MCC Ionogels: Concluding Remarks

In our study, we consider the triple BMImAc/Hal/MCC ionogel as a quasisolid material with increased elasticity, capable of self-healing. Guo et al. [18] reported on the possibility of using similar ionogels based on the IL of EMImAc for supercapacitors manufacturing. 

However, the following advantages of the BMImAc/Hal/MCC ionogel in comparison with the EMImAc/Hal/Cel system can be noted:

- lower freezing point of IL: <−20 °C (for BMImAc) and >30 °C (for EMImAc) [34];
- cellulose solubility in BMImAc is 2.5 times higher than in EMImAc [34];
- cellulose dissolves in BMImAc at a lower temperature than in EMImAc [34];
- the BMImAc/Hal/MCC ionogel has a conductivity close to that of the EMImAc/Hal/Cel ionogels (at a high temperature of 80 °C) and a much higher conductivity (approximately by 10 times) at low temperatures.

Thus, the triple BMImAc/Hal/MCC ionogels synthesised in current study can be judged promising materials for the use in flexible electronic devices and energy storage devices manufacturing.

## 3. Materials and Methods

### 3.1. Materials

To prepare ionogels, the following reagents were used without additional purification: halloysite nanotubes (Sigma-Aldrich, New York, NY, USA), 1–3 µm in length and 30–70 nm in diameter; microcrystalline cellulose, Avicel type, with an average size of 20 μM (Sigma-Aldrich, Bethesda, MD, USA); ionic liquid 1-butyl-3-methylimidazolium acetate, BMImAc (Sigma-Aldrich, Darmstadt, Germany, purity ≥ 95%, water content ≤ 2.0%).

BMImAc was chosen as a component of the ionogel because it is considered an effective cellulose solvent [35].

### 3.2. Preparation of the Ionic Liquid/Halloysite/Cellulose Ionogel

Triple IL/Hal/MCC ionogels were prepared according to the following two-step procedure. First, the cellulose was dried for 3 h at 60 °C. Then, it was dissolved in the ionic liquid (5 g) at 85 °C for 2 h using a magnetic stirrer. The concentration of MCC in the ionogel was 1, 2, 3 and 4 wt. %.

At the second stage, halloysite (2.5 g) was dispersed in IL/cellulose solution, using a vibrating shaker IKA VORTEX 4 (IKA-Werke GmbH and Co. KG, Staufen, Germany). Then, the mixture was sonicated in a CT-431D2 ultrasonic bath (CTbrand Wahluen Electronic Tool Co. Ltd., Shantou, China) for 2 h and then dried in an LT-VO/20 vacuum drying oven (Labtex, Moscow, Russia) at 80 °C for 24 h. The resulting ionogels were centrifuged for 45 min at 6000 rpm, and the excess of the ionic liquid was removed.

Hereafter, we use the abbreviations IL/Hal/MCC-1, IL/Hal/MCC-2, IL/Hal/MCC-3 and IL/Hal/MCC-4 to denote ionogels which were prepared using the IL/MCC systems with 1, 2, 3 and 4 wt. % of MCC, respectively, as precursors.

### 3.3. Methods

The microstructure of the IL/Hal/MCC ionogels was studied by scanning electron microscopy (SEM, Tescan Vega 3 SBH, the Czech Republic).

X-ray diffraction (XRD) patterns of the halloysite, BMImAc, MCC and resulting ionogels were investigated using a Bruker D8 Advance diffractometer (CuKα radiation, λ = 0.154 nm, Karlsruhe, Germany). The peak positions were calculated using the WinScaler 3.0 software.

The Fourier-transform infrared (FTIR) reflection spectra of the halloysite, BMImAc, MCC and synthesised ionogels were recorded using a VERTEX 80v infrared Fourier-transform spectrometer (Karlsruhe, Germany) in the region of 400–4000 cm^−1^ at room temperature and at a resolution of 2 cm^−1^**.**

Thermal analysis of BMImAc and ionogels was performed using a TG 209 F1 thermal analyser (NETZSCH, Selb, Germany). A sample (~10 mg) in a platinum crucible was heated from 25 to 800 °C in an argon flow. The heating rate was 10 °C/min. The accuracy of measuring the weight and temperature was ±10^–6^ g and ±0.1 °C, respectively. IL and ionogels were analysed three times to assess the reproducibility of the data.

Differential scanning calorimetry (DSC) thermograms of the ionic liquid and ionogels were recorded using a DSC 204 F1 Phoenix calorimeter (NETZSCH, Selb, Germany). The sample (10 mg) was placed in a sealed platinum crucible and then heated to 100 °C, cooled with liquid nitrogen to –110 °C and again heated to 100 °C. The cooling and heating rates were 10 °C/min. The measurements were carried out in an argon atmosphere. The accuracy of the temperature measurements was ±0.1 °C.

A Solartron SI 1260A Impedance/Gain-Phase analyser (Solartron Analytical, Hampshire, UK) was used to determine specific conductivity (*κ*). The measurements were performed in a hermetic cell using platinum electrodes. The cell temperature was maintained using a liquid cryothermostat LOIP FT-316-40 (LOIP, St. Petersburg, Russia) with an accuracy of ±0.2 °C.

In order to study the mechanical properties of the ionogels, a special test stand was used [36]. After mixing, ionogels were placed in the gap between the plunger and the press platform, and by moving the plunger at a rate of 0.0035 mm/s, the distance between them was brought to 5 mm. In this case, the ionogel filled the entire volume in the press gap.

## 4. Conclusions

For the first time, triple ionic liquid/halloysite/microcrystalline cellulose ionogels containing 1-butyl-3-methylimidazolium acetate were prepared using the two-stage synthesis, and their physicochemical properties were investigated. New data were obtained on changes in the properties of an ionic liquid upon intercalation into clay (a confinement effect) in the presence of a polymer. It was found that conductivity, as well the characteristic temperatures of phase transitions and the thermal decomposition of the BMImAc/Hal/MCC ionogels, depends on the percentage of components. The authors believe that halloysite and cellulose, in appropriate proportions, can be used as promising fillers for the development of high-performance ionogels, and the results of the research will be an important step on the path towards novel ion-conducting devices.

## Figures and Tables

**Table 1:** XRD data of IL/Hal/MCC ionogel samples.

Sample	2θ_0_ (Degree)	d_001_ (nm)
Hal	11.90	7.44
IL/Hal/MCC-1	11.99	7.38
IL/Hal/MCC-2	12.06	7.34
IL/Hal/MCC-3	11.96	7.40
IL/Hal/MCC-4	12.15	7.28

**Table 2:** The effect of cellulose content on phase-transition temperatures for BMImAc/MCC composites.

MCC Content (wt. %)	*T*_onset_ (°C)	*T*_g_ (°C)	*T*_end_ (°C)
0	−76.3	−72.5	−72.2
1	−71.9	−69.3	−68.6
2	−72.1	−68.7	−67.4
3	−78.8	−74.1	−73.1
4	−81.0	−78.1	−75.2

**Table 3:** The effect of cellulose content on the characteristic phase-transition temperatures for the BMImAc/Hal/MCC ionogels.

Sample	*T*_onset_ (°C)	*T*_g_ (°C)	*T*_end_ (°C)
IL/Hal (without MCC)	−63.8	−60.0	−59.3
IL/Hal/MCC-1	−77.9	−75.6	−74.3
IL/Hal/MCC-2	−80.0	−76.8	−75.6
IL/Hal/MCC-3	−74.2	−70.8	−70.3
IL/Hal/MCC-4	−84.7	−80.4	−78.0

**Table 4:** Characteristic temperatures and weight-loss values for the individual components of BMImAc/Hal/MCC ionogels.

Parameter	Hal	BMImAc	MCC
First stage			
*T*_1_ (°C)	42.9	73.4	40.6
*T*_d_ (°C)	55.9	102.1	53.2
*T*_2_ (°C)	83.7	137.2	80.8
Δ*m* (%)	2.1	5.1	4.1
Second stage			
*T*_1_ (°C)	432.3	220.6	316.3
*T*_d_ (°C)	478.1	242.3	337.2
*T*_2_ (°C)	507.9	259.3	352.0
Δ*m* (%)	12.9	94.0	95.4

**Table 5:** Characteristic temperatures and weight loss values for IL/Hal/MCC ionogels.

Parameter	IL/Hal Ionogel	IL/Hal/MCC Ionogel			
		IL/Hal/MCC-1	IL/Hal/MCC-2	IL/Hal/MCC-3	IL/Hal/MCC-4
**First stage**					
*T*_1_ (°C)	56.3	74.8	77.4	67.3	63.0
*T*_d_ (°C)	82.7	115.9	118.8	97.2	93.9
*T*_2_ (°C)	117.4	143.9	152.6	133.7	126.4
Δ*m* (%)	11.3	7.9	6.6	7.5	7.8
**Second stage**					
*T*_1_ (°C)	215.7	221.8	227.2	220.5	217.9
*T*_d_ (°C)	239.1	243.2	247.6	242.6	242.1
*T*_2_ (°C)	255.1	260.1	262.3	258.8	259.0
Δ*m* (%)	47.1	54.5	49.7	61.7	55.0
**Third stage**					
*T*_1_ (°C)	433.0	453.2	461.4	439.8	437.3
*T*_d_ (°C)	468.1	484.8	499.7	467.1	470.5
*T*_2_ (°C)	490.4	508.8	523.7	498.2	495.8
Δ*m* (%)	7.6	7.1	6.8	8.4	7.1

**Table 6:** Values of the specific conductivity of the ionogels.

Temperature (°C)	Specific Conductivity (S/m)		
	BMImAC	BMImAc/Hal	BMImAc/Hal/MCC-2
−30	0.0005	0.0038	0.0010
+80	1.1722	1.9926	1.2967

## Data Availability

Not applicable.

## Supplementary Materials

The following are available online. Figure S1: The dependence of the shear stress on the shear rate (a) and the dependence of apparent dynamic viscosity on the shear rate (b) for IL/Hal and IL/Hal/MCC-2 ionogels.

## Sample Availability

Samples of the compounds are available from the authors.
