# Supplementary material for: Enhancing the Thermal Stability of Ionogels: Synthesis and Properties of Triple Ionic Liquid/Halloysite/MCC Ionogels
Source: Molecules. 2021 Oct 14;26(20):6198. doi: 10.3390/molecules26206198 (PMC8538352; doi:10.3390/molecules26206198)
Supplement: Supplementary file 1 [file molecules-26-06198-s001.zip › molecules-1390823-supplementary.pdf]

Supplementary

Enhancing the Thermal Stability of Ionogels: Synthesis and Properties of Triple Ionic Liquid/Halloysite/MCC Ionogels

Olga V. Alekseeva [a], Valeriya D. Shibaeva [a], Andrew V. Noskov [a 1], Vladimir K. Ivanov [b], Alexander V. Agafonov [a]

[a] *G.A. Krestov Institute of Solution Chemistry, Russian Academy of Sciences, 153045 Ivanovo, Russia*

[b] *Kurnakov Institute of General and Inorganic Chemistry, Russian Academy of Sciences, 119991 Moscow, Russia*

The rheological properties of ionogels were measured using a DV-II+Pro viscometer (Brookfield, USA) in a continuous rotation mode and with different shear rates. The ionogel flow curves are shown in Figures S1a and b.

Analysis of the compression curves and dynamic shear behaviour of ionogels shows that the addition of cellulose to the IL/Hal ionogel causes some decrease in its compressive strength, while increasing shear stress and apparent viscosity (η). The observed phenomenon is associated with the formation of non-rigid bonds of cellulose molecules with clay particles, which leads to the formation of an elastic network that prevents flow under tangential loading but contributes to greater mobility of the structure under uniaxial compression. Thus, the introduction of MCC dissolved in BMImAc into the composition of the ionogel with halloysite makes it possible to regulate its viscoplastic properties, while maintaining high thermal stability.

[1] Corresponding author. E-mail: avn@isc-ras.ru (A.V. Noskov)

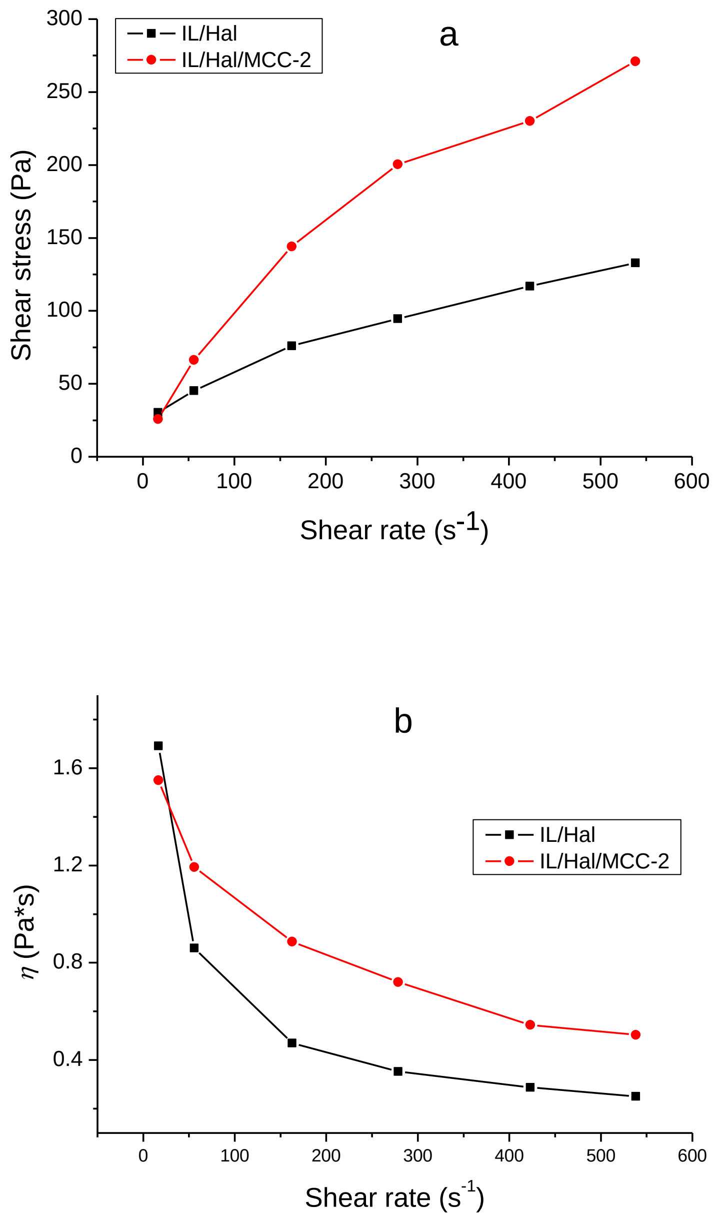

Figure S1. The dependence of shear stress on shear rate (a) and the dependence of apparent dynamic viscosity on shear rate (b) for IL/Hal and IL/Hal/MCC-2 ionogels
